# Curcumin Inhibits In Vitro SARS-CoV-2 Infection In Vero E6 Cells through Multiple Antiviral Mechanisms

**DOI:** 10.3390/molecules26226900

**Published:** 2021-11-16

**Authors:** Damariz Marín-Palma, Jorge H. Tabares-Guevara, María I. Zapata-Cardona, Lizdany Flórez-Álvarez, Lina M. Yepes, Maria T. Rugeles, Wildeman Zapata-Builes, Juan C. Hernandez, Natalia A. Taborda

**Affiliations:** 1 Grupo Infettare, Facultad de Medicina, Universidad Cooperativa de Colombia, 050012 Medellín, Colombia; bleidy1122@gmail.com (D.M.-P.); jorgetabare@gmail.com (J.H.T.-G.); wildeman.zapatab@campusucc.edu.co (W.Z.-B.); juankhernandez@gmail.com (J.C.H.); 2 Grupo Inmunovirología, Facultad de Medicina, Universidad de Antioquia, UdeA, 050010 Medellín, Colombia; mariaisab5@gmail.com (M.I.Z.-C.); liz.1.florez@gmail.com (L.F.-Á.); linayepes123@gmail.com (L.M.Y.); maria.rugeles@udea.edu.co (M.T.R.); 3 Grupo de Investigaciones Biomédicas Uniremington, Programa de Medicina, Facultad de Ciencias de la Salud, Corporación Universitaria Remington, 050016 Medellín, Colombia

**Keywords:** curcumin, antiviral, COVID-19, SARS-CoV-2, immune response, inflammation, D614G strain, Delta variant

## Abstract

Due to the scarcity of therapeutic approaches for COVID-19, we investigated the antiviral and anti-inflammatory properties of curcumin against SARS-CoV-2 using in vitro models. The cytotoxicity of curcumin was evaluated using MTT assay in Vero E6 cells. The antiviral activity of this compound against SARS-CoV-2 was evaluated using four treatment strategies (i. pre–post infection treatment, ii. co-treatment, iii. pre-infection, and iv. post-infection). The D614G strain and Delta variant of SARS-CoV-2 were used, and the viral titer was quantified by plaque assay. The anti-inflammatory effect was evaluated in peripheral blood mononuclear cells (PBMCs) using qPCR and ELISA. By pre–post infection treatment, Curcumin (10 µg/mL) exhibited antiviral effect of 99% and 99.8% against DG614 strain and Delta variant, respectively. Curcumin also inhibited D614G strain by pre-infection and post-infection treatment. In addition, curcumin showed a virucidal effect against D614G strain and Delta variant. Finally, the pro-inflammatory cytokines (IL-1β, IL-6, and IL-8) released by PBMCs triggered by SARS-CoV-2 were decreased after treatment with curcumin. Our results suggest that curcumin affects the SARS-CoV-2 replicative cycle and exhibits virucidal effect with a variant/strain independent antiviral effect and immune-modulatory properties. This is the first study that showed a combined (antiviral/anti-inflammatory) effect of curcumin during SARS-CoV-2 infection. However, additional studies are required to define its use as a treatment for the COVID-19.

## 1. Introduction

Since March 2020, the World Health Organization (WHO) has declared COVID-19 as a pandemic [1]. The severe acute respiratory syndrome coronavirus 2 (SARS-CoV-2), is an enveloped virus consisting of a positive-sense single-stranded RNA genome [2]. According to phylogenetic analysis, SARS-CoV-2 is a new member of the *Betacoronavirus* genus [3,4,5], which can infect humans and diverse animal species [6]. This virus is highly pathogenic and mainly affects respiratory tract cells, causing respiratory disease that can develop into severe, even life-threatening, pathologies. The SARS-CoV-2 genome encodes nonstructural, structural, and accessory proteins [6], which play essential roles within the viral replicative cycle [7,8,9]. Spike protein, 3C-like protease (3CLpro), papain-like cysteine protease (PLpro), and RNA-dependent RNA polymerase (RdRp) are the main antiviral targets [10].

After the virus enters the target cell, multiple interactions between viral components and host proteins triggers recognition by the immune system. Indeed, in severe COVID-19 cases, the uncontrolled viral replication induces aberrant pro-inflammatory responses, thus contributing to tissue damage [11,12]. According to this, it has been reported that UCI patients displays higher level of cytokines in plasma such as IL-2, IL-7, IL-10, G-CSF, IP10, MCP-1, MIP-1α, and TNF-α, compared with non-UCI patients [13]. Also, this relationship between unbalanced production of cytokines and COVID-19 pathogenesis has been described throughout transcriptomic analysis from COVID-19 patients, which revealed an excessive production of cytokines such as MCP-1, IP-10, MIP-1α, and MIP-1β [14].

Despite the unprecedented advances in the development of vaccines to prevent SARS-CoV-2 infection, their availability is still limited in some countries worldwide, making it necessary to search for alternative therapeutic strategies to control and reduce morbidity and mortality in COVID-19 patients. Besides, there are not enough data available about the effectiveness of therapeutic strategies with the current and upcoming variants. Thus, it is necessary to search for new therapeutic approaches.

Currently, several authors have suggested the potential of phytochemical compounds in the treatment against SARS-CoV-2 infection, which may prevent the onset of COVID-19 or its severity [15,16]. Among these, curcumin, the main polyphenolic compound of turmeric, has attracted significant attention owing to its biological effects, such as anti-tumor, anti-inflammatory, immunomodulating, antioxidant, antimicrobial, and antiviral activities [17]; therefore, it has been proposed that curcumin may be a potential treatment against COVID-19 [18]. Notably, it has been observed that the consumption of curcuminoids leads to a significant reduction in circulating levels of C-reactive protein [19] and decreases the expression of proinflammatory cytokines, including IL-1β, IL-6, IL-8, and TNF-α, demonstrating its anti-inflammatory capacity [20]; it is important to highlight that this cytokines has been correlated with severe illness. Moreover, in macrophages, it has been observed that curcumin inhibits NLR family pyrin domain containing 3 (NLRP3) inflammasome activation [21], which plays a significant role in the development of inflammatory diseases [22].

Additionally, curcumin exhibits its inhibitory activity against the replication of diverse viruses, such as dengue virus, hepatitis B virus, zika virus, influenza A virus, and chikungunya virus [23,24]. Curcumin can exert antiviral effects directly on the viral particle or at different stages of the replicative cycle by interacting with viral proteins or by modulating cellular processes or pathways crucial for viral replication [25,26,27]. Specifically for SARS-CoV-2, studies in silico (computer modelling) have reported that curcumin exhibits favorable binding affinities with the spike protein of the virus, as well as with its main cellular receptor, ACE2 [28]. These results suggest that curcumin has the ability to interfere with the entry of the virus into the cell. Furthermore, it has been reported that curcumin can affect the expression of other key molecules in the entry and decay of the virus, such as TMPRS22, Cat B, and L [29,30]. The cumulative evidence suggests that curcumin could be an effective treatment strategy to complement the COVID-19 clinical management.

Although curcumin has shown broad antiviral activity [26], and its potential as a treatment during COVID-19 has been proposed [27,28]. No studies have yet been done to test these hypotheses. For that, in this study, we investigated the antiviral and anti-inflammatory properties of curcumin against D614G strain and Delta variant of SARS-CoV-2 using in vitro models.

## 2. Results

### 2.1. Cytotoxicity of Curcumin on Vero E6

**Figure 1:**
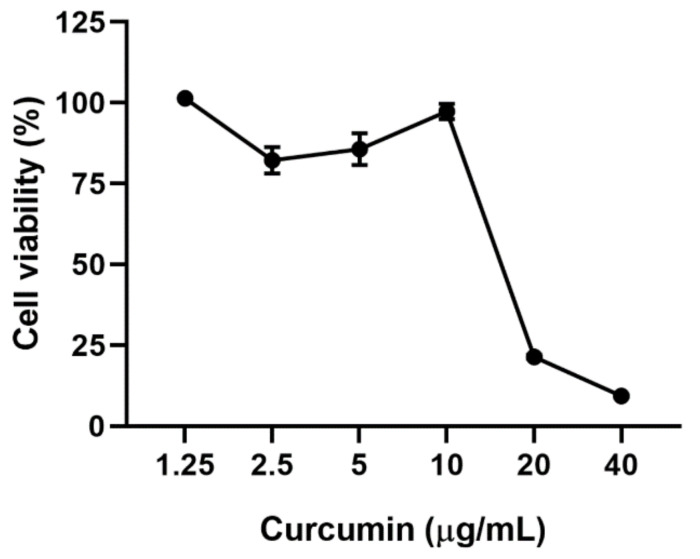
Cytotoxicity of curcumin on Vero E6. Viability of Vero E6 after 48 h of curcumin treatment (from 1.25 to 40 µg/mL). Data were presented as Mean ± SEM. The viability percentages of the treated cell were calculated based on untreated control (n = 8).

**Figure 2:**
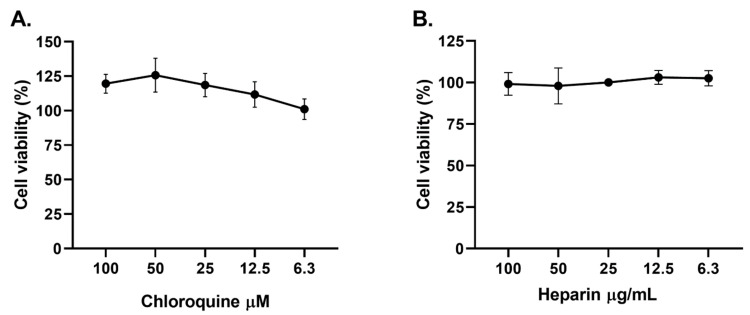
Positive controls of viral inhibition showed low cytotoxicity on Vero E6. The figure represents the viability percentage of Vero E6 cells after 48 h of treatment with (**A**) CQ (6.3–100 µM) and (**B**) heparin (6.3–100 µg/mL). Bars represent mean values ± SEM. Two independent experiments with four replicates each experiment was performed (n = 8).

Curcumin was cytotoxic at 20 and 40 µg/mL (cell viability of 21.4% and 9.34%, respectively) after 48 h of treatment on Vero E6. Contrarily, the viability of Vero E6 was higher or equal to 80% at curcumin concentrations of 10 µg/mL or lower (Figure 1). The CC50 (50% cytotoxic concentration) obtained for curcumin was 16.5 µg/mL. Positive controls of viral inhibition (chloroquine and heparin) did not affect cell viability at the evaluated concentrations (Figure 2).

### 2.2. Curcumin Inhibited the Early and Late Stages of SARS-CoV-2 D614G Strain

**Figure 3:**
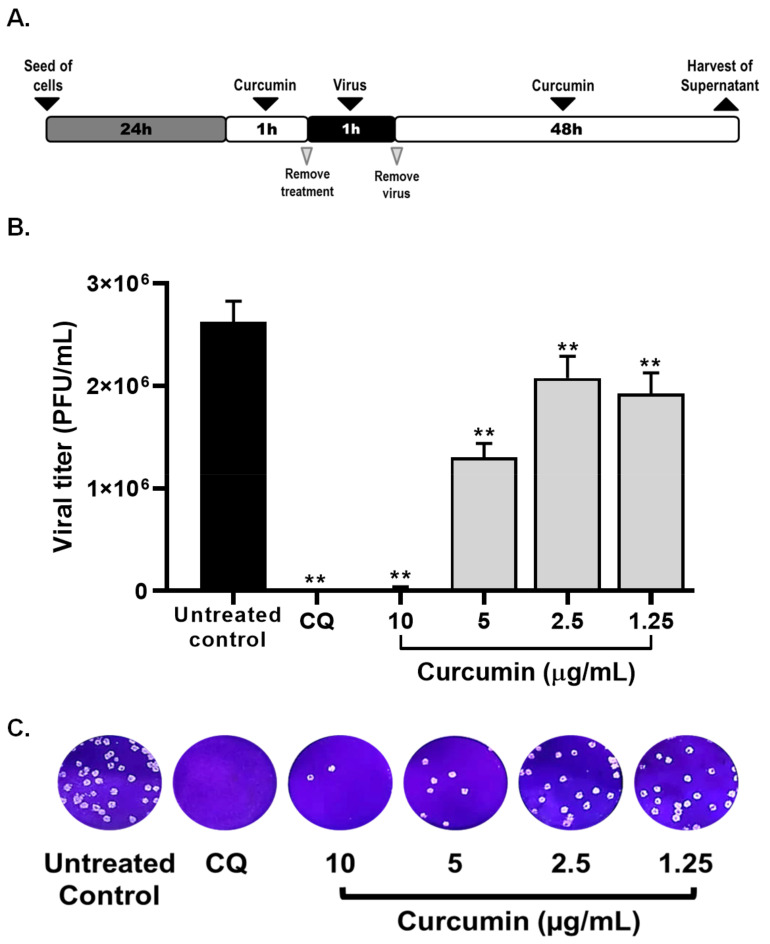
Antiviral effect of curcumin against SARS-CoV-2 by pre–post infection treatment. (**A**) Representative scheme of pre–post infection treatment. (**B**)The figure shows the reduction of D614G strain titer (PFU/mL) on Vero E6 supernatants after pre–post infection treatment with curcumin (n = 4). Chloroquine (CQ) was included as a positive control of viral inhibition. Data were presented as median ± IQR (interquartile range). Mann–Whitney test ** *p*  ≤  0.01. Inhibition percentages of 99%, 51.3%, 22.2%, and 27.8% were obtained at 10, 5, 2.5, and 1.25 µg/mL of curcumin, respectively. (**C**) Representative plaques on Vero E6 cells of pre–post infection treatment of curcumin against D614G strain.

The antiviral effect of non-cytotoxic concentrations of curcumin (1.25–10 µg/mL) were evaluated through four treatment strategies and using a MOI of 0.01. By pre–post infection treatment (cells treatment with curcumin, prior and post to infection with SARS-CoV-2) curcumin exerted antiviral activity of 99.0% (*p* = 0.0095), 51.3% (*p* = 0.0095), 22.2% (*p* = 0.0095), and 27.8% (*p* = 0.0095) against SARS-CoV-2 D614G strain at concentrations of 10, 5, 2.5, and 1.25 µg/mL, respectively (Figure 3). An EC50 (50% maximal effective concentration) of 4.06 µg/mL (3.09–5.16 µg/mL) was calculated for curcumin, with an SI (Selectivity Index) of 4.06 (Table 1), by pre–post infection treatment. An inhibition of 100% (*p* = 0.0095) was observed for chloroquine treatment (positive control of viral inhibition) (Figure 3).

**Figure 4:**
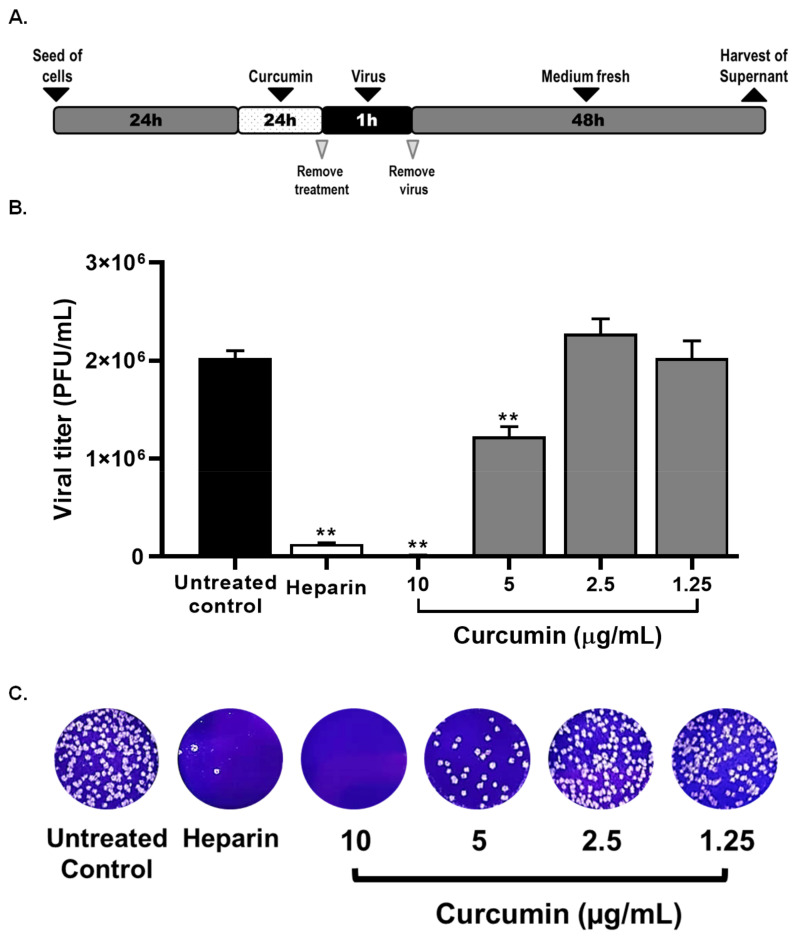
Curcumin inhibited SARS-CoV-2 by pre-infection treatment strategy. (**A**) Representative scheme of pre-infection treatment. (**B**) The figure represents the reduction of D614G strain titer (PFU/mL) on Vero E6 supernatants after pre-treatment with curcumin (from 1.25 to 10 µg/mL). Heparin was used as a positive control of viral inhibition. Data were presented as median ± IQR (n = 4). Mann–Whitney test ** *p*  ≤  0.01. Inhibition percentages of 99.2%, 39.3%, −12.8%, and −0.4% were obtained at 10, 5, 2.5, and 1.25 µg/mL of curcumin, respectively. (**C**) Representative plaques on Vero E6 cells of pre-treatment of curcumin against D614G strain.

Then, the pre-infection and post-infection treatment strategies were performed to identify the steps of the SARS-CoV-2 replicative cycle affected by curcumin. The pre-infection treatment demonstrated that curcumin had an antiviral effect against SARS-CoV-2 D614G strain at 10 µg/mL of 99.2%, *p* = 0.0095 and at 5 µg/mL of 39.3%, *p* = 0.0095 (Figure 4). Differences in viral titer were not observed at 2.5 and 1.25 µg/mL of curcumin. The EC50 value calculated for curcumin was 5.02 µg/mL, with an SI of 3.29, by the pre-infection treatment.

**Figure 5:**
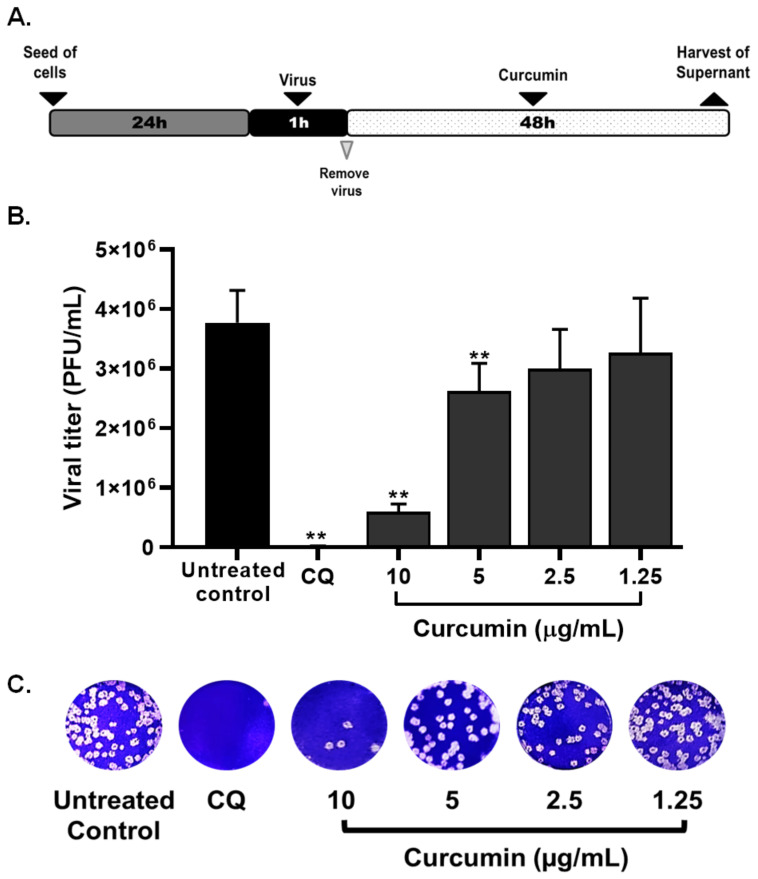
Curcumin inhibited the SARS-CoV-2 by post-infection treatment strategy. (**A**) Representative scheme of post-infection treatment. (**B**) The figure represents the reduction of D614G strain titer (PFU/mL) on Vero E6 supernatants after post-infection treatment with curcumin (from 1.25 to 10 µg/mL). Chloroquine (CQ) was included as a positive control of viral inhibition. Data were presented as median ± IQR (n = 4). Mann–Whitney test ** *p*  ≤  0.01. Inhibition percentages of 84.4%, 31.7%, 21.9%, and 14.8% were obtained at 10, 5, 2.5, and 1.25 µg/mL of curcumin, respectively. (**C**) Representative plaques on Vero E6 cells of post-infection treatment of curcumin against D614G strain.

After the post-infection treatment, curcumin exerted an antiviral effect against SARS-CoV-2 D614G strain at 10 µg/mL of 84.4%, *p* = 0.0095, and at 5 µg/mL of 31.7%, *p* = 0.0095 (Figure 5). However, inhibitions of 21.9% and 14.8% were observed at 2.5 and 1.25 µg/mL of curcumin, respectively. The EC50 value calculated for curcumin was 6.03 µg/mL (4.83–7.41 µg/mL), with an SI of 2.74, by the post-infection treatment.

Heparin and chloroquine were the positive controls for viral inhibition in the pre-infection and post-infection treatments, respectively. From the pre-infection treatment, an inhibition percentage of 87.6% (*p* = 0.0095) was obtained at 25 µg/mL of heparin (Figure 4), whereas an inhibition of 99.3% (*p* = 0.0095) was obtained by post-infection treatment with chloroquine (Figure 5).

### 2.3. Curcumin Inhibited SARS-CoV-2 D614G Strain Infectivity under the Co-Treatment Condition

**Figure 6:**
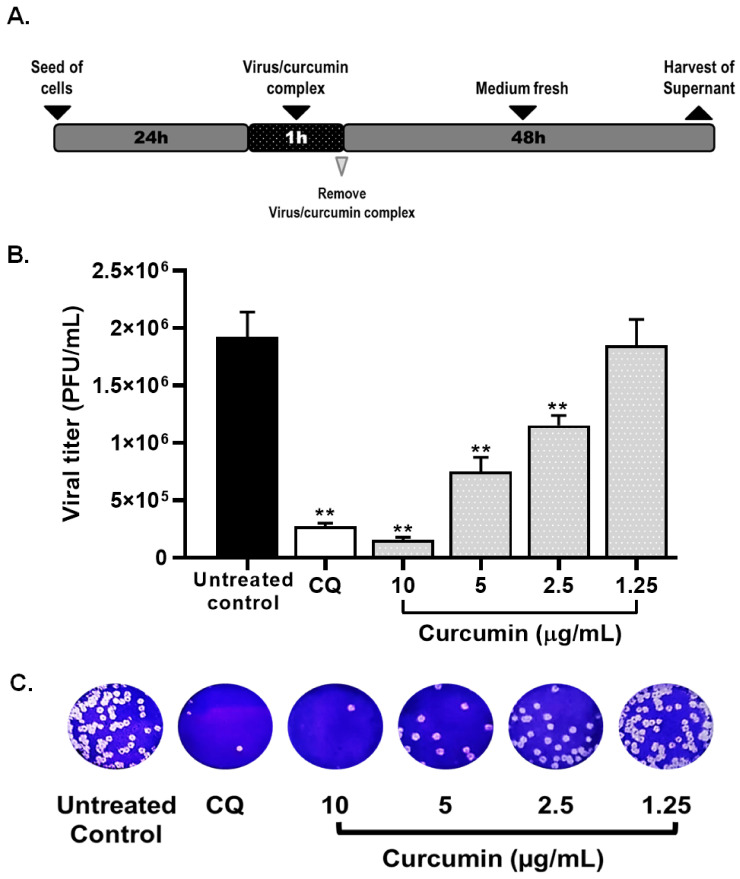
Curcumin inhibited SARS-CoV-2 infectivity under the co-treatment condition. (**A**) Representative scheme of co-treatment strategy. (**B**) The figure represents the reduction of D614G strain titer (PFU/mL) on Vero E6 supernatants after co-treatment treatment with curcumin (from 1.25 to 10 µg/mL). Chloroquine (CQ) was used as a positive control of viral inhibition. Data were presented as median ± IQR (n = 4). Mann–Whitney test ** *p*  ≤  0.01. Inhibition percentages of 92%, 60.4%, 39.3%, and 2.3% were obtained at 10, 5, 2.5, and 1.25 µg/mL of curcumin, respectively. (**C**) Representative plaques on Vero E6 cells of the co-treatment strategy of curcumin against SARS-CoV-2 D614G strain.

As can be seen from Figure 6, the viral titer of D614G strain was significantly reduced through co-treatment strategy (incubation of curcumin with the virus prior to infection) at 10 (92%, *p* = 0.004), 5 (60.4%, *p* = 0.004), and 2.5 µg/mL (39.3%, *p* = 0.004) of curcumin (Figure 6). An inhibition of 2.3% was obtained at 1.3 µg/mL. An EC50 of 3.57 µg/mL (3.06–4.17 µg/mL) was calculated for curcumin, with an SI of 4.62, from co-treatment. Finally, an inhibition percentage of 83% (*p* = 0.004) was observed by co-treatment with chloroquine (positive control of viral inhibition) (Figure 6).

### 2.4. Treatment with Curcumin Also Exhibed the Infection by SARS-CoV-2 Delta Variant

**Figure 7:**
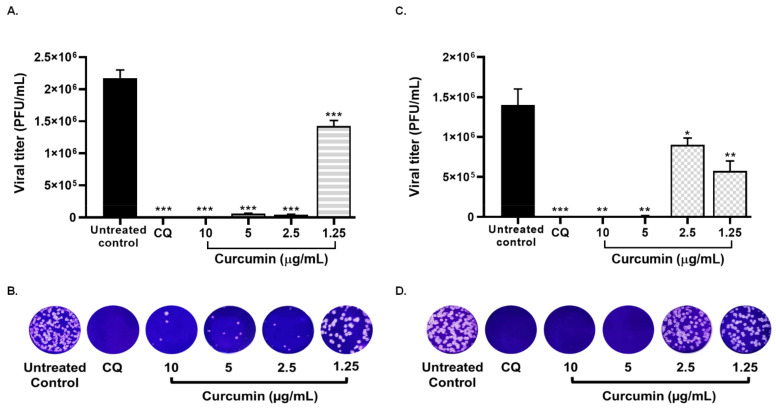
Treatment with curcumin inhibited the infection by SARS-CoV-2 Delta variant. (**A**) The figure represents the reduction of Delta variant titer (PFU/mL) on Vero E6 supernatants after pre–post infection treatment with curcumin (from 1.25 to 10 µg/mL). Inhibition percentages of 99.8%, 98.4%, 98.9%, and 62.9% were obtained at 10, 5, 2.5, and 1.25 µg/mL of curcumin, respectively. (**B**) Representative plaques on Vero E6 cells of the pre–post infection strategy of curcumin against SARS-CoV-2 Delta variant. (**C**) The figure shows the reduction of Delta variant titer (PFU/mL) on Vero E6 supernatants after co-treatment with curcumin (from 1.25 to 10 µg/mL). Inhibition percentages of 99.9%, 99.1%, 31.9%, and 56.5% were obtained at 10, 5, 2.5, and 1.25 µg/mL of curcumin, respectively. (**D**) Representative plaques on Vero E6 cells of the co-treatment of curcumin against SARS-CoV-2 Delta variant. Chloroquine (CQ) was used as a positive control of viral inhibition. Data were presented as median ± IQR (n = 4). Mann–Whitney test ** *p*  ≤  0.01, * *p*  ≤  0.05**.** *** *p* ≤  0.001.

Considering that curcumin showed inhibition in all treatment strategies, this compound was evaluated against infection by the SARS-CoV-2 Delta variant using pre–post infection and co-treatment. As shown in the Figure 7A,B, curcumin inhibited Delta variant at 10 µg/mL (99.8%, *p* = 0.0007), 5 (98.4%, *p* = 0.0007), 2.5 (98.9%, *p* = 0.0007), and 1.25 µg/mL (62.9%, *p* = 0.0007), by pre–post infection treatment (EC50 = 1.14 µg/mL, SI = 14.5). In addition, curcumin showed antiviral activity of 99.9% (*p* = 0.0012), 99.1% (*p* = 0.0012), 31.9% (*p* = 0.0233), and 56.5% (*p* = 0.0017) against Delta variant, at concentrations of 10, 5, 2.5, and 1.25 µg/mL, respectively, using a co-treatment strategy. The EC50 value calculated for curcumin was 1.66 µg/mL, with an SI of 9.94, by the co-treatment (Figure 7C,D). These results indicated that the anti-SARS-CoV-2 effect of curcumin was not dependent on the infecting strain/variant. Chloroquine (positive control of viral inhibition) showed antiviral activity against the Delta variant using pre-post infection treatment (100%, *p =* 0.0007) and co-treatment (100%, *p =* 0.0002) (Figure 7).

### 2.5. Curcumin Showed Anti-Inflammatory Effects in PBMCs Challenged with SARS-CoV-2

**Figure 8:**
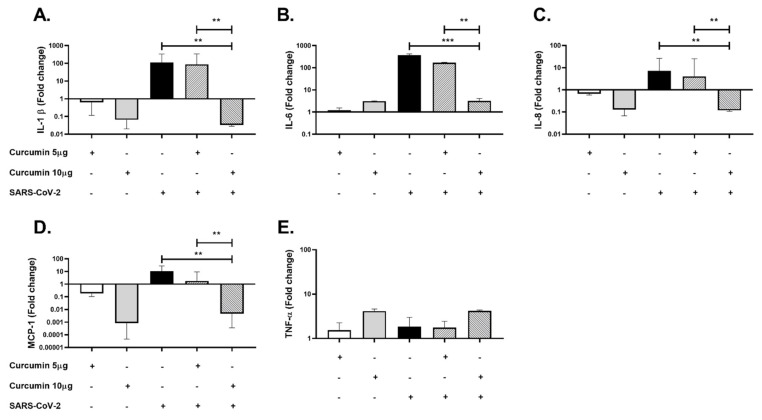
Anti-inflammatory effect of curcumin in PBMCs stimulated with SARS-CoV-2. Gene expression of Inflammatory cytokines was quantified in PBMCs by real-time PCR. The figure represents the fold change of (**A**) IL-1β, (**B**) IL-6, (**C**) IL-8, (**D)** MCP-1, and (**E)** TNF-α. Cells untreated were used as a negative control. Data were represented as median ± IQR (n = 6). Mann–Whitney test ** *p*  ≤  0.01, *** *p*  ≤  0.001.

To evaluate the potential anti-inflammatory effect of curcumin on SARS-CoV-2 infection, PBMCs were pretreated with curcumin and stimulated with SARS-CoV-2 at 0.1 MOI in 50 µL of RPMI supplemented with 5% FBS for 24 h. After, the cells and supernatants were collected for cytokine (mRNA and protein) quantification. Significant decreases in IL-1β mRNA (*p* = 0.0022, Figure 8A), IL-6 mRNA (*p* < 0.001, Figure 8B), IL-8 mRNA (*p* = 0.0022, Figure 8C), and MCP-1 mRNA (*p* = 0.0050) were found in PBMCs pretreated with 10 µg/mL of curcumin compared with cells stimulated only with the virus. No significant changes in the mRNA expression of TNF-α was found (Figure 8D).

**Figure 9:**
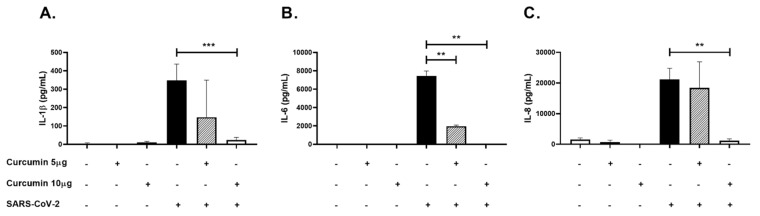
Anti-inflammatory effect of curcumin in PBMCs stimulated with SARS-CoV-2. Inflammatory cytokines were quantified in PBMCs supernatants by ELISA. The figure represents the concentrations of (**A**) IL-1β, (**B**) IL-6, and (**C**) IL-8. Cells untreated were used as a negative control. Data were represented as median ± IQR (n = 6). Mann–Whitney test ** *p*  ≤  0.01, *** *p* ≤  0.001.

Similarly, a decrease in IL-1β (*p* < 0.0001, Figure 9A), IL-6 (*p* = 0.0022, Figure 9B) and IL-8 (*p* = 0.0022, Figure 9C) protein levels determined by ELISA was observed in the supernatant from PBMCs pretreated with 10 µg/mL of curcumin compared to cells stimulated only with the virus.

## 3. Discussion

There is still no conclusive information on the effectiveness of approved drugs for COVID-19 treatment, therefore, the discovery of effective and nontoxic antiviral agents is still necessary. Curcumin has shown a wide range of antiviral activity against different viral models [31]. Similar to these reports, our findings indicated that curcumin inhibits SARS-CoV-2 D614G strain which contains the most widespread amino acid change (D614G in the spike protein) carried by more than 99% of the prevalent variants since the beginning of 2020 [32,33]. According to previous reports, curcumin exhibited moderate selectivity for pre-infection and post-infection treatment strategies. Contrarily, low selectivity was obtained for pre–post infection treatment and co-treatment strategies [34].

Curcumin inhibited SARS-CoV-2 D614G strain by pre-infection treatment of Vero E6 cells. This effect has also been observed with other enveloped viruses such as Influenza, Dengue, Zika, Chikungunya, Japanese encephalitis, Pseudorabies, and Vesicular stomatitis virus [24,35,36], showing that curcumin treatment affects the early stages of the replicative cycle, such as viral attachment, internalization, fusion, or decapsidation [37]. With regard to SARS-CoV-2, spike protein binds to its human receptor ACE2 (angiotensin-converting enzyme 2) through its receptor-binding domain [28,29]. Previous studies have reported a favorable binding affinity of curcumin to the spike protein (−7.9 Kcal/mol) and its cell receptor, ACE2 (angiotensin-converting enzyme 2) (−7.8 Kcal/mol) [28]. According to the above, it could be suggested that curcumin prevents the recognition of the target cell and subsequent SARS-CoV-2 entry by direct interaction with cell factors or viral proteins [28,38,39]. This effect could be related to our results obtained by co-treatment which suggest a possible virucidal activity of curcumin against SARS-CoV-2 D614G strain.

Thus, this compound may directly interfere with structural glycoproteins or other viral envelope components that affect viral infectivity, as previously reported for Influenza virus [35,40] and respiratory syncytial virus. Furthermore, treatment with curcumin could induce changes in the morphology of the SARS-CoV-2 viral particle, as reported for Murine norovirus-1 [41]. However, further studies are necessary to confirm the antiviral mechanism of this compound.

On the other hand, the membrane fusion stage requires the cleavage of the spike protein by cellular proteases [42]. This process occurs through the activation of TMPRSS2 (transmembrane serine protease 2) on the cell membrane or by the priming cathepsin B/L in the endosomes [29,30,43]. This last route depends on pH [29,30]. With respect to this, a study reported that curcumin inhibits cellular protease TMPRSS2 (Trans-Membrane Serine Protease 2), which cleaves to the S2 subunit of spike protein to reveal the fusion peptide [29,39]. In addition, it was demonstrated that this compound inhibits vacuolar-ATPase expression, which acts as a proton pump during the acidification of vesicles [44]. According to these studies, the mechanism of anti-SARS-CoV-2 activity of curcumin could involve the inactivation of cell enzymes participating in viral fusion with host membranes, thus blocking viral entry.

The post-infection treatment results indicates that curcumin can effectively inhibit SARS-CoV-2 D614G strain up to 87%, affecting the post-entry steps of the viral replicative cycle. Previously, it has been reported that curcumin inhibits SARS-CoV at a range of 3–10 µM, similar to our results [45]. It has also been reported that curcumin has a specific inhibitory effect on 3CL protease observed using the FRET method with a reported IC50 of 40 µM [45]. Furthermore, the antiviral activity of curcumin has been reported for other viruses, such as respiratory syncytial virus, where curcumin inhibits replication and budding, and HIV, where curcumin was shown to interact with active sites of protease and integrase [46,47]. In the case of SARS-CoV-2, docking analysis has shown that curcumin has low binding energies and inhibition constants [48], suggesting that this might be a potential mechanism of curcumin antiviral effect observed in this study. For example, it has been shown that curcumin exhibited multiple interactions with the Nsp9 replicase, which can directly affect viral replication [49].

Taking into account that curcumin exhibited inhibition against D614G strain, through different treatment strategies, this compound was evaluated against infection by the Delta variant [50] which contains mutations in the spike protein (L452R, T478K, P681R, and D614G) associated with an increase in viral infectivity, transmissibility and pathogenicity in individuals infected with SARS-CoV-2 and a decrease in antibody-mediated neutralization [50]. Similar to that reported for PRRSV (porcine reproductive and respiratory syndrome virus) [51], our results showed that the antiviral effect of curcumin is virus strain/variant independent. In this context, curcumin inhibited the Delta variant up to 99.9%, exhibiting higher selectivity than the obtained for D614G strain, through pre–post infection and co-treatment strategies. This finding is particularly important because antiviral drugs that are aimed at a conserved viral target could not only help reduce the possibility of the disease progressing to serious illness, but could also be used as a prophylactic strategy, helping to solve the problem of reduced response of variants to vaccines [52].

According to the results obtained in vitro, we suggest the importance of evaluating the antiviral activity of curcumin in other cell lines, such as Calu-3 or A549 cells transfected with human ACE2 gene [53], which are permissive for SARS-CoV-2 infection [53].

The hyperactivation of immune cells and anomalous release of cytokines play a foremost role in poor outcomes in several viral diseases, including COVID-19. Such a systemic production of cytokines is often referred to as a cytokine storm [54]. This elevation in cytokine levels has been linked as the culprit behind deterioration and multiple organ failure in COVID-19 [55]. In this study, curcumin-treated PBMCs stimulated with SARS-CoV-2 exhibited a reduction in the production and release of pro-inflammatory cytokines, including IL-1β, IL-6, MCP-1, and IL-8. Similar results have been reported for COVID-19 patients treated with nano-curcumin (nano-micells or nano-particles formulation to encapsulate curcumin) [56]. The anti-inflammatory capacity of curcumin and its potential for use in treating viral infections, including coronavirus infections, have been hypothesized previously [56]. Activation of C-terminal leucine-rich repeats and NLRP3 inflammasome through downstream signaling of Toll-like receptor (TLRs) encourages maturation and release of proinflammatory cytokines. Moreover, TLRs activate NF-κB, which prompts the transcriptional activation of cytokines and other inflammatory molecules [57]. Therefore, previous reports indicate the downregulation of TLRs by curcumin [58]. In addition to TLRs, it has been shown that curcumin can diminish NF-κB activation [59], as well as inhibit the NLRP3 inflammasome [60], which could play a significant role in the development and progression of COVID-19.

Further, it has been persistently reported that curcumin has anti-inflammatory effects on in vivo models, such as atherosclerosis, multiple sclerosis, Alzheimer’s, or arthritis [56,61,62,63,64,65]. These studies demonstrated that curcumin blocks inflammation in parts by preventing the activation of macrophages and lymphocytes and inhibiting the production of pro-inflammatory cytokines and chemokines [65,66,67]. In this sense, it has been shown that despite the low bioavailability of curcumin, in two models of chronic disease, this compound has anti-inflammatory effects at low doses, via IL-10 production [68].

Moreover, the ability of curcumin to alter the inflammatory state through the modulation of its regulatory elements can prevent the onset of the cytokine storm. The modulation of the cytokine release in SARS-CoV-2-infected patients can be crucial in the prevention of severe disease. Evidence presented in this article suggests that curcumin represents a promising compound for developing therapy against SARS-CoV2.

In this study, curcumin showed high cytotoxicity at 20 µg/mL in Vero E6 cells. However, the above minimal toxicity has been reported for this compound at doses of up to 8000 mg in humans [69]. This evidence shows that the toxicity obtained from these compounds through in vitro assays does not always overlap with that obtained from in vivo evaluations [70]. These differences could be related to the exposure time and supplementation with other compounds for their administration in humans [70,71]. Based on the above, our study enables us to make an approximation to the effect of this compound as an antiviral; however, it is important to evaluate the toxicity of curcumin when its antiviral effect is determined through in vivo models to ensure the safety of this compound.

In conclusion, curcumin showed in vitro antiviral activity against SARS-CoV-2, with different treatment strategies, which suggest the inhibition at different stages of the replicative cycle; furthermore, these effects seem to be independent of the virus strain/variant. This antiviral effect, together with the observed immunomodulatory properties, suggests that curcumin could be a promising compound for the treatment of COVID-19 patients. However, complementary studies are necessary to establish its efficacy in animal and human models, as well as its mechanisms of action.

## 4. Materials and Methods

### 4.1. Cells and Virus

*Cercopithecus aethiops* kidney cell line Vero E6 was grown in Dulbecco′s Modified Eagle Medium (DMEM, Sigma-Aldrich, St. Louis, MO, USA) supplemented with 2% heat-inactivated fetal bovine serum (FBS) (GIBCO), 1% penicillin–streptomycin (GIBCO), and 2 mM L-glutamine (Sigma-Aldrich) at 37 °C with 5% CO_2_. With three passages per week. Vero E6 cells were donated by Instituto Nacional de Salud, Bogotá-Colombia (Dr. José Usme, 11 April 2020). Infections were carried out with viral stocks produced from two Colombian SARS-CoV-2 isolates: D614G strain (EPI_ISL_536399) [72] and Delta variant (EPI_ISL_5103929).

### 4.2. Curcumin Stock Preparation

Curcumin was purchased from MERCK (Item No. C1386; Darmstadt, Germany). Curcumin powder was solubilized in dimethyl sulfoxide (DMSO; Sigma, D-2650, Darmstadt, Germany) at 10 mg/mL. The stock was maintained at 4 °C protected from light until use. The working solution was prepared, diluting the stock at 40 µg/mL in DMEM supplemented with 2% FBS.

### 4.3. Cytotoxicity Assay

The cytotoxicity of curcumin on Vero E6 cells was evaluated using an MTT assay [73]. Briefly, Vero E6 cells were seeded in 96-well plates at a density of 1.0 × 10^4^ cells/well in DMEM supplemented with 2% FBS. The plates were incubated for 24 h at 37 °C with 5% CO_2_. After incubation,100 µL/well of serial double dilutions of curcumin ranging from 1.25 to 40 µg/mL was added to cell monolayers and incubated for 48 h. Subsequently, the supernatant was removed, the cells were washed twice with phosphate buffered saline (PBS) (Lonza, Basel, Switzerland), and 30 µL/well of MTT (2 mg/mL) was added. After the addition of MTT, the plates were incubated for 2 h at 37 °C, with 5% CO_2,_ protected from light. Finally, 100 µL/well of DMSO was added. Plates were read at 570 nm using a Multiskan^TM^ GO Microplate Spectrophotometer (Thermo-Scientific, Waltham, MA, USA). The average absorbance of untreated cells was used as viability control. The cell viability of each treated well was calculated based on the viability control. Concentrations with cell viability after treatment of 80% or more were considered non-cytotoxic. For the MTT assay, two independent experiments with four replicates each were conducted (n = 8).

### 4.4. Evaluation of the Antiviral Activity against SARS-CoV-2

The antiviral activity of curcumin was initially evaluated using a pre–post infection treatment strategy [74]. Briefly, Vero E6 cells were seeded in 96-well plates (1.2 × 10^4^ cells/well) and incubated for 24  h, at 37 °C with 5% CO_2_. Then, serial double dilutions of curcumin (from 1.25 to 10 µg/mL) were prepared and added to cell monolayers (50 µL/well) for 1 h. After pretreatment, the curcumin-containing medium was aspirated from cell monolayers, and the virus was added at 0.01 MOI (multiplicity of infection) in 50 µL of DMEM supplemented with 2% FBS. After 1 h of viral adsorption at 37 °C, the inoculum was removed and replaced by 150 µL/well of the same pre-treatment dilutions. Finally, the plates were incubated for 48 h at 37 °C under a 5% CO_2_ atmosphere.

To determinate which steps of the viral cycle are being affected by curcumin treatment, the antiviral activity was evaluated using three extra strategies: (i) Co-treatment: Serial double dilutions of curcumin were mixed with SARS-CoV-2 (MOI 0.01) and incubated for 1 h at 37 °C (1:1 ratio). After incubation, 50 µL/well of the virus-treatment mixture was added to cell monolayers and incubated at 37 °C for 1 h. After 1 h of viral adsorption, the mixture (curcumin–virus) was removed and replaced by 150 µL/well of fresh medium. The plates were incubated for 48 h at 37 °C with 5% CO_2_. (ii) Pre-infection treatment: 150 µL/well of serial double dilutions of curcumin was added to cell monolayers for 24 h before viral infection. (iii) Post-infection treatment: 150 µL/well of serial double dilutions of curcumin was added to cell monolayers for 48 h after infection. In all cases, two independent experiments with four replicas each were performed (n = 8). Chloroquine (100 µM) [75] or heparin (25 µg/mL) [76] were used as positive controls of viral inhibition.

### 4.5. Quantification of Antiviral Activity by Plaque Assay

The viral titer of antiviral assay supernatants was determined by plaque assay. Briefly, 1.2 × 10^5^ Vero E6 cells/well were seeded in 24-well plates for 24 h, at 37 °C, with 5% CO_2_. Subsequently, 10-fold serial dilutions of the supernatant obtained from all antiviral assays (200 µL/well) were added to cell monolayers and incubated for 1 h at 37 °C with 5% CO_2_. Then, the viral inoculum was removed and replaced with 1 mL of semi-solid medium (1.5% carboxymethyl-cellulose in DMEM 1× with 2% FBS and 1% Penicillin–Streptomycin). The cells were incubated for 3 d at 37 °C and then washed twice with PBS and fixed-stained with 4% Formaldehyde/1% crystal violet solution; then, the viral plaques were counted. The difference between viral titer after curcumin treatment and untreated control was expressed as an inhibition percentage. Two independent experiments with two replicates each were conducted (n = 4).

### 4.6. Evaluation of Anti-Inflammatory Activity in PBMCs Stimulated with SARS-CoV-2

Approximately 20 mL of total peripheral blood from each healthy adult donor (n = 3) was obtained by venipuncture in vacutainer tubes. PBMCs were isolated using the Ficoll-Histopaque density gradient method (Sigma-Aldrich Chemical Co., St. Louis, MO, USA) as previously described [77].

To evaluate the anti-inflammatory effect of curcumin, the PBMCs were seeded in 24-well plates (1 × 10^6^ cells/well) in RPMI1640 medium (Sigma-Aldrich) supplemented with 5% FBS. The cells were stimulated with curcumin (5 and 10 µg/mL) for 1 h. After pre-treatment, the SARS-CoV-2 was added at a MOI of 0.1 and incubated for 24 h, at 37 °C with 5% CO_2_. Three independent experiments with two replicates were conducted (n = 6). The cells without any treatment were used as negative controls.

### 4.7. RNA Extraction, cDNA Synthesis, and Real-Time PCR

The mRNA quantification for IL-1β, IL-6, IL-8, MCP-1, and TNF-α was carried out in PBMCs by real-time polymerase chain reaction (real-time PCR) as previously described [78]. Briefly, for total RNA extraction, the Direct-zol RNA Miniprep kit was used (Zymo Research, Orange, CA, USA). RNA concentration/purity were determined by spectrophotometry at 260–280 nm and cDNA was synthesized with 140 ng of RNA using the iScript cDNA synthesis kit (BIO-RAD, Hercules, CA, USA), according to the manufacturer’s instructions.

Real-time PCR was performed using Maxima SYBR Green qPCR master mix kit (Fermentas, Glen Burnie, MD, USA). The mRNA PGK (phosphoglycerate kinase) was used as the housekeeping gene to normalize the RNA content (Table 2). The amplification protocols were 40 cycles and standardized for each gene. For real-time RT-PCR analysis, the CFX Manager Version: 1.5.534.0511 software (Bio-Rad, Hercules, CA, USA) was used. Data are expressed as fold change, normalized against the constitutive gene and the untreated control, using the ΔΔCt method, as previously reported [79].

### 4.8. ELISA

Concentrations of IL-1β, IL-6, and IL-8 were quantified in PBMCs culture supernatants using ELISA kits (#437004, Biolegend, Thermofisher; #555220, BD Biosciences, San Jose, CA, USA; and #431504 Biolegend, Thermofisher, respectively) and according to the manufacturer’s instructions.

### 4.9. Statistical Analysis

All data were analyzed with GraphPad Prism (La Jolla, CA, USA). Data were presented as median ± IQR (interquartile range) or mean ± SEM (standard error of the mean), as indicated in each figure. Statistical differences were evaluated by Student′s t-test or Mann–Whitney U test based on Shapiro–Wilk normality test. A *p* value ≤ 0.05 was considered significant. The EC50 (50% effective concentration) and CC50 (50% cytotoxic concentration) values were determined by non-linear regression analysis using a sigmoidal model. The selectivity index (SI) was calculated from the CC50/EC50 ratio.

### 4.10. Ethics

This study was approved by the ethics committee of the Universidad Cooperativa de Colombia (Acta BIO106). It was carried out keeping good records, practicing good data collection and management, transparency of data-sharing, and realistic representation of study results. The data were analyzed anonymously. All donors were adults, read, and signed an informed consent. All research protocols were made according to the principles of the Declaration of Helsinki.

## Figures and Tables

**Table 1:** CC50, EC50, and SI values for curcumin in Vero E6 cells infected with SARS-CoV-2.

Compound	CC50 (µM)	Strain/Variant	Treatment Strategy	EC50 (µg/mL)	SI
Curcumin	16.5	D614G strain	Pre–post infection treatment	4.06	4.06
			Pre-infection treatment	5.02	3.29
			Post-infection treatment	6.03	2.74
			Co-treatment	3.57	4.62
		Delta variant	Pre–post infection treatment	1.14	14.5
			Co-treatment	1.66	9.94

**Table 2:** Primers sequence.

Gene	Sequence of Primers 5′−3′	*Annealing Temperature*
IL-1β	Fw: GGATATGGAGCAACAAGTGG Rv: ATGTACCAGTTGGGGAACTG	60 °C
IL-6	Fw: GGGGTGGTTATTGCATC Rv: ATTCGGTACATCCTCGAC	56 °C
IL-8	Fw: ACTGAGAGTGATTGAGAGTGGAC Rv: AACCCTCTGCACCCAGTTTTC	60 °C
TNF-a	Fw: GGCTCCAGGCGGTGCTTGTTC Rv: AGA-GGCGATGCGGCTGATG	60 °C
PGK (Housekeeping gene)	Fw: GTTGACCGAATCACCGACC Rv: CGACTCTCATAACGACCCGC	60 °C

## Data Availability

All data generated or analyzed during this study are included in this published article (and its supporting information files).

## Sample Availability

Samples of the compounds are available from the authors.
